# NTRK Fusions Detection in Paediatric Sarcomas to Expand the Morphological Spectrum and Clinical Relevance of Selected Entities

**DOI:** 10.3389/pore.2022.1610237

**Published:** 2022-02-28

**Authors:** Filippo Nozzoli, Alexander J. Lazar, Francesca Castiglione, Domenico Andrea Campanacci, Giovanni Beltrami, Francesco De Logu, Chiara Caporalini, Daniela Massi, Giandomenico Roviello

**Affiliations:** ^1^ Section of Anatomic Pathology, Department of Health Sciences, University of Florence, Florence, Italy; ^2^ Departments of Pathology and Genomic Medicine, The University of Texas M.D. Anderson Cancer Center, Houston, TX, United States; ^3^ Department of Orthopaedic Oncology and Reconstructive Surgery, Careggi University Hospital, Florence, Italy; ^4^ Department of Paediatric Orthopaedic Oncology, Meyer Children’s Hospital, Florence, Italy; ^5^ Clinical Pharmacology and Oncology Unit, Department of Health Sciences, University of Florence, Florence, Italy; ^6^ Pathology Unit, Meyer Children’s Hospital, University of Florence, Florence, Italy; ^7^ Medical Oncology, Department of Health Sciences, University of Florence, Florence, Italy

**Keywords:** Pan-Trk, NTRK, ETV6-NTRK3, NTRK-rearranged tumors, paediatric sarcomas

## Abstract

Undifferentiated round cell sarcomas (URCS) of soft tissue and bone and tumours of uncertain differentiation (TUD) are commonly ascribed to a subset of neoplasms with low frequency of NTRK gene fusions. However, more recently NTRK-rearranged round and spindle cell tumours have been noted in case reports and in limited or heterogeneous cohorts. The aim of our study was to investigate the presence of NTRK gene fusions in a large retrospective cohort of paediatric URCS and TUD after a systematic review of the diagnosis, according to the recently updated WHO classification scheme. One-hundred and five patients with diagnosis of URCS or TUD, involving the bone or soft tissue, were retrospectively evaluated. After the case selection and the histopathological review of the case cohort, pan-Trk immunohistochemistry (IHC) testing was performed on formalin-fixed paraffin-embedded (FFPE) tissues. Tumour RNA was extracted from FFPE tissue and subjected to next-generation sequencing (NGS) library preparation, using a 10-gene NGS fusion panel, sequenced on an Illumina MiSeq. The NGS-positive cases were further confirmed by real-time PCR. On immunohistochemical screening, 12/105 (11.4%) cases were positive using the pan-Trk antibody, showing three different staining patterns with the cytoplasmic distribution being most common. Molecular analysis using NGS and confirmed by the real-rime PCR detected two positive cases for the ETV6-NTRK3 fusion. The histological pattern of the two positive cases, together with the demonstration of the NTRK rearrangement, leaded to re-classify these previously not otherwise specified sarcomas with uncertain differentiation into the emerging category of NTRK-rearranged neoplasms. In addition, we found the two NTRK fused neoplasms showing a clinical indolent course, in contrast with literature.

## Introduction

According to the 2020 WHO Classification of Soft Tissue and Bone Tumours (1), the category of undifferentiated round cell sarcomas (URCS) includes a new section containing not only the paradigmatic round cell sarcoma, well known as Ewing sarcoma, but also three distinct entities with different clinical, pathological, and molecular features: round cell sarcomas with EWSR1 gene fusion to non-ETS family members, CIC-rearranged sarcomas, and BCOR-rearranged sarcomas. When no identifiable line of differentiation is shown when analysed by presently available technology, the classification scheme allows to include these entities within the heterogeneous group of undifferentiated soft tissue sarcomas (USTS). The detection of gene rearrangements in URCS and USTS represents a crucial challenging field of diagnostic pathology, as the tumour molecular profiling turned out to have a crucial role in establishing accurate classification schemes and correspondingly in improving the quality of diagnosis and therapeutic options. URCS and USTS are commonly ascribed to a category of neoplasms with low frequency of NTRK gene fusions (2). The inclusion of NTRK-rearranged spindle cell neoplasms (excluding infantile fibrosarcoma that represent a distinct clinicopathologic entity molecularly characterized by the presence of NTRK3-ETV6 fusion gene), within the category of tumours of uncertain differentiation represents one of the most relevant innovations. Most recently, NTRK-rearranged round cell sarcomas have been noted in heterogeneous case studies (3–5). In the largest study conducted on NTRK fusions by Solomon et al. (6), including multiple detection assays across more than 33,000 cases of a wide range of tumour types, immunohistochemistry for pan-Trk staining demonstrated positivity for 1 of 5 Ewing sarcomas and 3 of 5 sarcomas with BCOR translocations, but no positivity for NTRK gene fusions was found by RNA sequencing. Specifically, all immunohistochemically positive cases demonstrated cytoplasmic or membranous expression only, and no nuclear staining was observed. In a cohort of 30 paediatric NTRK-rearranged mesenchymal tumours, Davis et al. (7) characterized the clinicopathologic features of 12 classic ETV6-NTRK3 fused infantile fibrosarcoma and 18 variant paediatric NTRK-rearranged mesenchymal tumours. This series included a TPM3-NTRK1 rearranged inflammatory spindle and round cell sarcoma and an ETV6-NTRK3 rearranged spindle and round cell sarcoma. The aim of our retrospective study was to detect NTRK fusions in a cohort of more than 100 paediatric cases of undifferentiated round cell sarcoma and tumours with uncertain differentiation, following the published ESMO guidelines on methods and diagnostic algorithm to target NTRK fusion (8). In our scenario, NTRK fusions needed to be screened in a population where prevalence of such gene fusions was not expected at high frequency, and a targeted sequencing assay was available, therefore we followed the recommendation to adopt a two-step approach: IHC followed by targeted RNA sequencing, with the latter representing the gold standard for molecular detection. We also aimed to demonstrate the utility of the detection methods used in this family of neoplasms. Prior to the immunohistochemical and molecular testing, we re-examined the entire case collection to assess the diagnosis in the light of the diagnostic criteria of the 2020 WHO Classification of Soft Tissue and Bone Tumours.

## Materials and Methods

### Ethics Approval

The use of FFPE samples of human tissue was approved by the Regional Ethics Committee for Clinical Trials of Tuscany (15248_bio). This study was performed in accordance with the Declaration of Helsinki. Written informed consent has been obtained from the patients to publish this paper.

### Case Selection and Tumour Specimen Collection

A total of 105 paediatric patients, aged between 1 and 21 years old, diagnosed with first diagnosis of round cell sarcoma of soft tissue or bone or tumour of uncertain differentiation, observed and treated at Careggi University Hospital (Florence, Italy) and Meyer Children University Hospital (Florence, Italy) between 2000 and 2020, were included. The haematoxylin and eosin (HE) stained slides and formalin-fixed paraffin-embedded (FFPE) tissue specimens were selected in the archive of the Pathology Unit, Careggi University Hospital (Florence, Italy). The histologic diagnosis was based on the results of open biopsy and/or surgical excision. Prior to NTRK immunohistochemistry and molecular testing, all cases were re-reviewed to confirm the diagnosis, to exclude those re-qualified as doubts, those related to post-chemotherapy treatment or exposed to decalcifying agents. If needed, additional immunohistochemical and/or FISH analyses were performed to support the final diagnosis. Eligibility criteria to enter this study also included a tumor content of >50% per slide and the lowest possible rate of fibrosis and necrosis.

### Clinical Data Collection

Clinical features were considered for age, sex, presentation, treatment, and outcome. Staging was reported with both the main systems currently proposed: the Enneking (9) classification and the UICC/AJCC classification (10), with the latter more informative as it considers the skips metastases and the different metastases pattern. The tumour size was determined by CT scan measurement of the three diameters of the and the tumour volume was calculated with the following formula: AxBxC, where dimensions are expressed as width (A) by length (B) by depth (C) in three-dimensional space. The response to neoadjuvant chemotherapy was determined by histologic examination, according to the Picci method (11) and consequently classified as grade 1, for evidence of macroscopic foci of viable tumour cells; grade 2, when only isolated microscopic nodules of viable tumour cells can be observed; grade 3 to indicate absence of nodules of viable cells. This staging method was simplified labelling the histological response as “good” in the case of grade 2 or 3, “poor” in case of grade 1. Other clinical features taken into account included the presence of metastases at diagnosis, local and distant recurrences, and oncologic outcomes (CDF, continuous disease free; NED, no evidence of disease; AWD, alive with disease; DOC, dead other cause; DOD, dead of disease). The follow up data were considered for 95 of the 105 total cases, as 10 cases had too recent diagnosis for follow up data to be collected.

### Morphology, Immunoprofile and Molecular Review

The entire specimen collection was reviewed for the morphology features and immunoprofile to re-examine the diagnosis, according to the 2020 WHO Classification of Soft Tissue and Bone Tumours. HE stained sections and immunohistochemical stains, including those from the pathologic evaluation made at time of diagnosis, were revised by two independent pathologists and cases of difficult interpretation were discussed under the microscope until full consensus was achieved. A small round cell morphology and the CD99 membranous expression by immunohistochemistry were therefore considered necessary to validate the diagnosis of Ewing sarcoma, with the EWSR1-ETS family group fusion detection as desirable criterion in selected cases. A spindled to rounded cytomorphology and the EWSR1 break-apart FISH showing amplification of the 5′ probe in EWSR-non-ETS rearrangement (EWSR1-NFATC2) were considered essential for the diagnosis of round cell sarcoma with EWSR1-non-ETS fusions (1). CIC-rearranged sarcoma needed predominant round cell phenotype, an immunoprofile showing variable CD99 staining, with WT1, ETV4 positivity and CIC gene rearrangement demonstration. The diagnosis of BCOR-rearranged sarcoma was assessed with primitive round to spindle cells arranged in nests, sheets, or fascicular growth; immunohistochemical positivity for BCOR, SATB2, cyclin D1 and a molecular confirmation of BCOR genetic abnormality as desirable criterion. The definition of undifferentiated sarcoma was applied with the following elements: spindle, pleomorphic, epithelioid, or round cell morphology; absence of any morphological or immunohistochemical feature of specific differentiation; demonstrated absence of distinctive molecular aberration.

### Immunohistochemistry

Immunohistochemistry was performed on 4 μm paraffin-embedded whole tissue sections using standard techniques. IHC staining for Trk A, B, and C expression was performed with pan-Trk antibody, a rabbit recombinant monoclonal antibody (mAb) clone EPR17341 (#790-7026, ready to use, Ventana Medical Systems, Tucson, AZ, United States). All assays were performed on Ventana Discovery XT immunostainer (Ventana Medical Systems, Tucson, AZ, United States), a fully automated method for staining and designed for the immunohistochemical detection of the C-terminal region of proteins A, B and C of tropomyosin receptor kinase (Trk), known to be preserved in fusion proteins both chimeric and “wild-type”. The staining protocol included pre-treatment with cell conditioner followed by incubation with antibody. The signal for antibody was then developed with Discovery anti-Rabbit HQ, Discovery Anti-HQ HRP and CromoMap DAB. After the staining run was complete, the tissue sections were counterstained with haematoxylin. The laboratory practice included a control tissue sample of infantile fibrosarcoma containing elements with positive and negative staining to use it as both positive and negative control. Pan-Trk IHC staining was assessed in both tumour nuclei and cytoplasm and recorded as weak, moderate, or strong, as previously described (12). Specifically, in the case of staining distributed only to the cytoplasm, the involvement of at least 50% of the neoplastic cells was required; in the case of nuclear involvement, any reactivity, even if minimal, was considered acceptable to assess the case as positive. Other patterns, such as perinuclear and membranous, were also assessed, as previously reported (12). Further IHC antibodies performed to support the final diagnosis are listed in Table 1.

**TABLE 1:** Summary of additional IHC antibodies performed to support the final diagnosis.

Antibody	Technology	Clone	Dilution
CD99	Rabbit Monoclonal	SP119	1:200
CD34	Mouse Monoclonal	QBEND/10	1:100
S-100	Rabbit Polyclonal	-	1:500
Desmin	Rabbit Monoclonal	SP138	1:100
Vimentin	Rabbit Monoclonal	SP20	1:200
SMA	Mouse Monoclonal	1A4	1:100
Pan Cytokeratin	Mouse Monoclonal	AE1/AE3	1:100
Ki-67	Rabbit Monoclonal	SP6	1:250
SOX10	Rabbit Monoclonal	EPR4007-104	1:250
Chromogranin A	Rabbit Polyclonal	-	1:200
Synaptophysin	Rabbit Monoclonal	SP11	1:200
BCOR	Rabbit Polyclonal	-	1:100
Pan Trk	Rabbit Monoclonal	EPR17341	1:500

### RNA Extraction and Quantification

The haematoxylin and eosin-stained tumor slides were examined to identify representative areas of tumor suitable for molecular testing. RNA extraction from 12 FFPE tumor specimens, previously characterized for IHC pan-Trk positivity, was carried out according to the manufacturers’ protocols, utilizing the MagCore® Total RNA FFPE One-Step Kit (RBC Bioscience Corp., New Taipei City, Taiwan) on the automated extraction system MagCore® Super (RBC Bioscience Corp., New Taipei City, Taiwan), based on magnetic beads extraction technology. RNA input quantification was measured by real-time PCR on EasyPGX® qPCR instrument 96 (Diatech Pharmacogenetics, Jesi, Italy), ensuring an accurate and precise measurement of the amplifiable RNA in the following amplification reaction of the target library regions. The assay allows the detection of two RNA regions highly conserved of 105 and 175 bp; the detection was performed using probes labelled with FAM and HEX, respectively. The RNA concentration was assessed by quantification with a standard curve in the HEX channel; the ratio between the quantification (ng/μl) obtained in FAM and that obtained in the HEX allows to evaluate the DNA fragmentation. The analysis of the assay was done by the dedicated EasyPGX® Analysis Software (Diatech Pharmacogenetics, Jesi, Italy), that calculates the concentration and degree of fragmentation of the samples. After the quantification step, of the original 12 cases selected for sequencing, two cases were respectively excluded due to insufficient quantity and quality of nucleic acids. Therefore, 10 tumor samples with adequate quantity and quality of nucleic acids for sequencing were utilized for NGS library preparation.

### RNA Libraries Preparation and NGS Sequencing

RNA libraries were generated using the Myriapod® NGS Cancer panel RNA (Diatech Pharmacogenetics, Jesi, Italy), according to the manufacturers’ instructions. The kit consists in a commercially available CE-IVD workflow from extraction to bioinformatics and it allows the detection of fusions regarding ten recurrently rearranged cancer genes: ALK, ROS1, RET, NTRK1, NTRK2, NTRK3, FGFR2, FGFR3, PPARG and the skipping of exon 14 of MET in total RNA, isolated from tumour tissue. The RNA was retro-transcribed into cDNA using random hexamers. Subsequently, cDNA was amplified by multiplex-PCR using two primer mixtures to obtain fragments between 47 and 184 bases, including fusions of interest and endogenous control genes (PCR1). The amplification products were purified with magnetic beads to remove residual primers. An amplification-based indexing reaction (PCR2) follows which allows a unique pair of two sample-specific barcodes (indexes) and an Illumina® platform-specific adapter to be attached to each fragment. The libraries thus constituted were normalized in quantity by magnetic beads to guarantee a homogeneous coverage of the samples during sequencing. Finally, the normalized libraries were mixed (library pool) and sequenced in parallel on the Illumina® MiSeq™ platform (Illumina Inc., San Diego, CA, United States) with MiSeq™ Reagent Kit v2 Micro (300 cycles) flow cell (Illumina Inc., San Diego, CA, United States).

### Sequencing Data Analysis

The data generated by the sequencer were then analyzed locally with the dedicated Myriapod® NGS Data Analysis Software (Diatech Pharmacogenetics, Jesi, Italy).

### Real-Time-PCR

Following a two-step approach, data obtained with NGS were confirmed by real-time PCR. The kit used was the “EasyPGX® ready NTRK fusion” (Diatech Pharmacogenetics, Jesi, Italy), a commercially available CE-IVD test for the detection of NTRK1, NTRK2 and NTRK3 gene fusions in the total RNA selected from FFPE tumour sample and amplified by One Step Real-Time RT-PCR. The control group comprised a control assay: an expressed gene amplified in the channel stably and independently from the original tumour tissue to verify the correct execution of the amplification process, the RNA quantity used and the possible presence of inhibitors that can cause false negative results. A positive and a negative control were also used. The detection took place via fluorescent probe marked with FAM and HEX; it is also composed of eight assays for the detection of gene alterations. Each of them provides the simultaneous detection of the target, through a probe marked with FAM, and an endogenous control gene, through a probe marked with HEX. Data were analyzed by EasyPGX® Analysis Software (Diatech Pharmacogenetics, Jesi, Italy).

## Results

### Morphology, Immunoprofile, Molecular Review

Our cohort consisted of 105 paediatric patients with suspected diagnosis of undifferentiated round cell sarcoma of soft tissue or bone. Of those, 96 cases were assessed as Ewing sarcoma, 3 as EWSR1-non-ETS round cells sarcoma, 1 as sarcoma with BCOR genetic alteration and 5 cases showed no identifiable line of differentiation and were labelled as USTS. The main clinical and pathologic features of the cohort are summarized in Table 2.

**TABLE 2:** Clinical and pathologic features of all the 105 patients included in the cohort.

Age	
<2	5
2–5	9
6–14	59
15–21	32
**Sex**	
Male	58
Female	47
**Site**	
Soft Tissue	23
Bone	82
**Volume**	
<100 cm^3^	72
>100 cm^3^	33
**Enneking Staging**	
I–II	71
III–IV	34
**UICC/AJCC Staging**	
I–II	59
III–IV	46
**Metastasis at diagnosis**	
Yes	23
No	82
**Treatment**	
Surgery	105
Neoadjuvant chemotherapy	105
**Response to chemotherapy**	
Poor (Picci grade 1)	32
Good (Picci grade 2-3)	63
Not Available	10
**DOD (dead of disease)**	
Yes	26
No	79
**CD99 expression**	
Yes	99
No	6
**t(11;22)(q24;q12) translocation**	
Yes	96
No	6
Not available	3

### Immunohistochemistry

**FIGURE 1:**
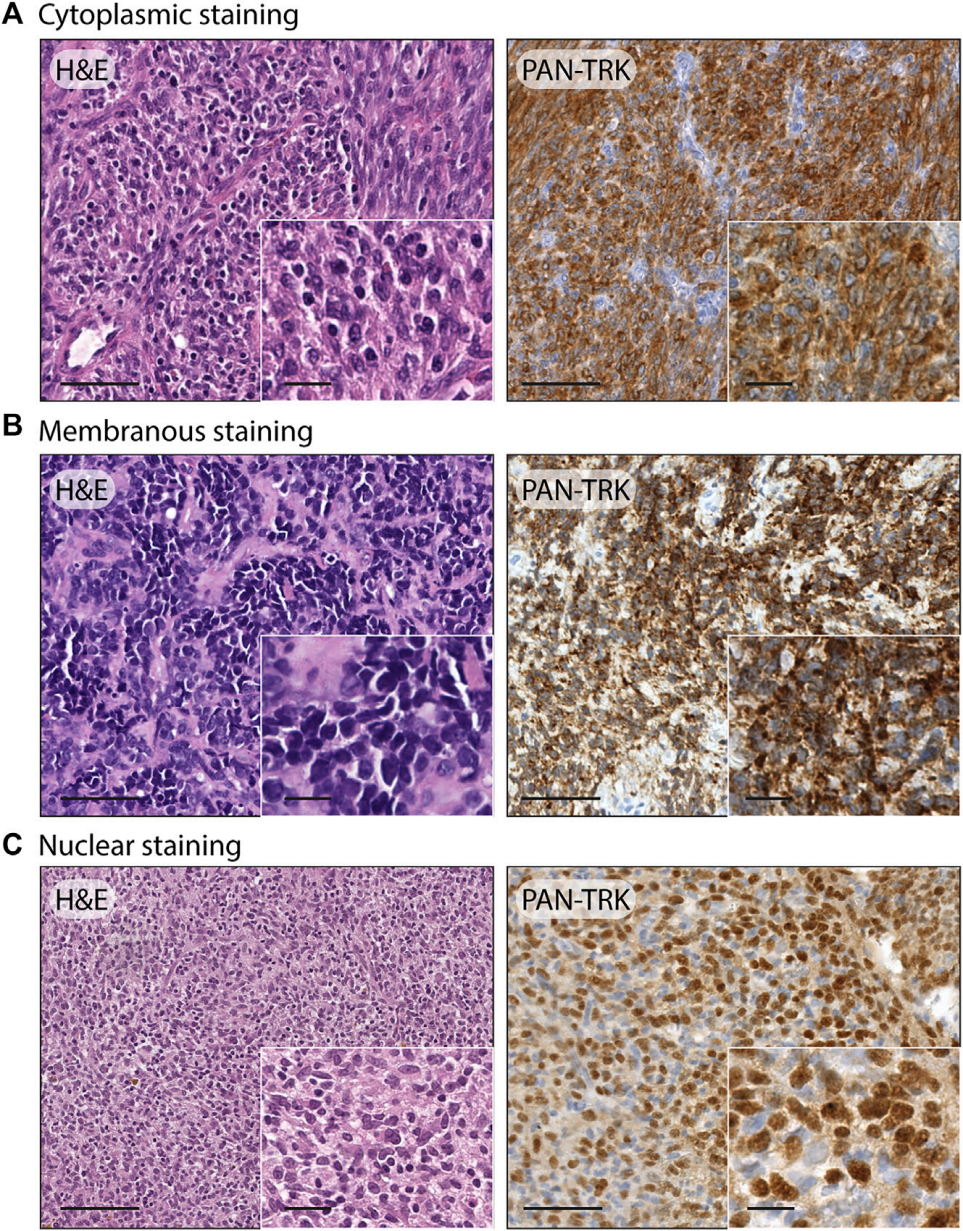
Examples of different staining patterns found in the case series. **(A)** Ewing sarcoma: H&E, 20x (scale bar = 100 μm) and 40× (scale bar = 25 μm); pan-TRK IHC with cytoplasmic staining, 20× (scale bar = 100 μm) and 40× (scale bar = 25 μm). **(B)** Ewing sarcoma: H&E, 20× (scale bar = 100 μm) and 40× (scale bar = 25 μm); pan-TRK IHC with membranous staining, 20× (scale bar = 100 μm) and 40× (scale bar = 25 μm). **(C)** Undifferentiated soft tissue sarcoma: H&E 20× (scale bar = 100 μm) and 40× (scale bar = 25 μm); pan-TRK IHC with nuclear staining, 20× (scale bar = 100 μm) and 40× (scale bar = 25 μm).

After the immunohistochemistry screening, 12/105 cases emerged for the Trk protein expression, including 8/96 Ewing sarcomas, 3/5 USTS and 1/1 sarcoma with BCOR genetic alteration, with a comprehensive percentage of positive immunoreactivity of 11.4%. The pan-Trk staining showed three different cellular distribution patterns, specifically: isolated cytoplasmic staining was the predominate pattern found, as it occurred in 9/12 cases (7 Ewing sarcoma, 1 USTS and 1 sarcoma with BCOR genetic alteration) while a nuclear staining in 2/12 cases (2 USTS) and a membranous staining in 1/12 cases (1 Ewing sarcoma). Examples of the three different cellular staining distribution patterns found are shown in Figure 1. Demographic, clinical, immunohistochemical, and molecular data of the twelve IHC pan-Trk positive cases are summarized in Table 3.

**TABLE 3:** Summary of clinical, immunohistochemical, and molecular data of the twelve IHC pan-Trk positive cases.

Pts	Age (years)	Sex	Diagnosis	Location	Mass volume (cm^3^)	AJCC staging	Oncologic outcome	IHC Pan-Trk pattern	NTRK fusion
1	14	M	ES	Lower Leg	12.6	IIB	DOD	Cytoplasmic	-
2	10	F	ES	Sacrum	0.6	IIA	CDFS	Cytoplasmic	-
3	13	M	USTS	Lower Leg	45	IIIA	CDFS	Nuclear	ETV6-NTRK3
4	10	F	ES	Iliac Crest	56	IIB	DOD	Membranous	-
5	11	M	USTS	Thigh	10.8	IIA	CDFS	Nuclear	ETV6-NTRK3
6	2	F	USTS	Lower Leg	3	IIA	NED	Cytoplasmic	-
7	13	M	ES	Lower Leg	28.2	IIIA	CDFS	Cytoplasmic	-
8	12	F	ES	Scapula	241.3	IVB	DOD	Cytoplasmic	-
9	11	F	ES	Sacrum	105.6	IVB	CDFS	Cytoplasmic	-
10	10	M	BCOR	Lower Leg	35	IIA	CDFS	Cytoplasmic	-
11	13	F	ES	Pelvis	12	IIA	CDFS	Cytoplasmic	*
12	16	M	ES	Upper Leg	52.5	IIB	CDFS	Cytoplasmic	*

*RNA, not suitable for testing.2.

ES, ewing sarcoma; USTS, undifferentiated soft tissue sarcoma; BCOR, sarcoma with BCOR, genetic alterations; DOD, dead of disease; CDFS, continuous disease-free survival; NED, no evidence of disease.

### Molecular Testing

**FIGURE 2:**
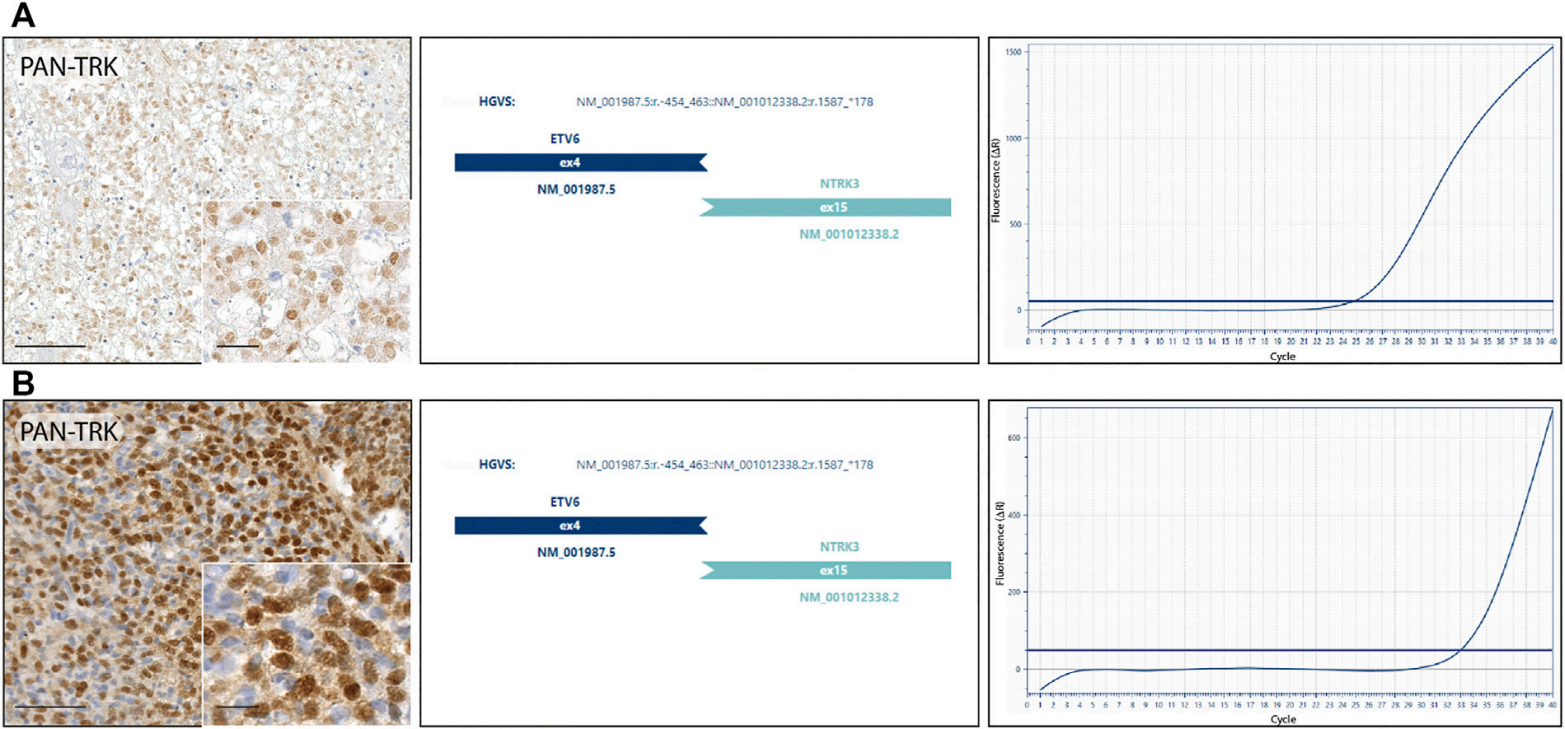
NTRK fusion tumours. **(A)** Undifferentiated soft tissue sarcoma: diffuse nuclear IHC pan-Trk staining with ETV6-NTRK3 fusion detected by next-generation sequencing and confirmed by real-time PCR. **(B)** Undifferentiated soft tissue sarcoma: strong nuclear IHC pan-Trk staining with ETV6-NTRK3 fusion detected by next-generation sequencing and confirmed by real-time PCR.

NGS RNA fusion panel testing was successfully completed for 10 cases, revealing ETV6-NTRK3 fusion in 2 tumor samples, confirmed by real-time PCR. Both cases harbouring NTRK fusion, showed a nuclear staining distribution pattern when the pan-Trk antibody was performed (Figure 2). Other 8 cases resulted negative for NTRK fusions, while the remaining 2 cases were not assessable because the RNA was not suitable for testing after the quantification process. The first NTRK fusion positive case (Figure 2A) is a 13-year-old male with an initial diagnosis of Ewing-like sarcoma involving the extraosseous soft tissue component along the left tibia, then re-diagnosed as undifferentiated soft tissue sarcoma. The tumour mass, calculated by the product of all the three diameters of the lesion, had a volume of 45 cm^3^ and presented itself as a IIB grade, according to the Enneking staging system and as a IIIA, according to the AJCC staging. Histopathologically, the neoplasm showed a polymorphic small round and spindle cell phenotype and a weak CD99 immunoexpression, lacking the canonical EWSR1-ETS family rearrangement demonstration; immunohistochemical findings also showed positivity for S-100 and pan-Trk with a nuclear distribution pattern. The pan-Trk IHC positivity was not known at the time of treatment. Neoadjuvant chemotherapy was given [ISG/SSG III protocol, using 6 drugs active in Ewing’s family tumours: Ifosfamide (Ifo), Etoposide (Eto), Vincristine (V), Actinomycin-D (Act), Adriamycin (Adm), Cyclophosphamide (C)]. The patient showed a Picci grade 3 response, meaning no evidence of viable tumour cells. After surgery, maintenance chemotherapy was adopted with VAdmC regimen as first cycle, then continued with the same 6 drugs and modalities as in the induction treatment. Without local relapse or distant metastases after surgery, the oncologic result is a continuous disease-free survival during the last 4 years. The second NTRK-fused tumour in our cohort (Figure 2B) belongs to a 11-year-old male with an expansive mass of 10.8 cm^3^ of volume, observed on the left thigh and diagnosed as undifferentiated soft tissue sarcoma. The open biopsy revealed a predominately spindle cells morphology with isolated round elements. The immunohistochemical profile included weak S-100, CD99, vimentin and desmin; pan-Trk testing showed a strong nuclear distribution pattern. Surgery for the tumour resection was performed as local treatment, and adjuvant multiagent chemotherapy (VAC/IE protocol: Vincristine + Doxorubicin + Cyclophosphamide, alternating with Ifosfamide + Etoposide) was then adopted, resulting in no further relapse with a continuous disease-free survival, persisting for the last 6 years.

## Discussion

The focus on NTRK rearrangements detection has increased recently due to some specific molecular targeted agents that demonstrated a marked and durable antitumour activity in patients with NTRK gene fusions positive cancers, regardless of tumour histology (13,14). This study evaluated the presence of NTRK gene fusions in a large paediatric cohort entirely composed of URCS and USTS of soft tissue and bone, where the cases were categorized, according to the 2020 WHO classification. The immunohistochemistry performed with pan-Trk antibody showed reactivity in 12/105 (11.4%) analyzed samples, but the molecular analysis showed positivity for ETV6-NTRK3 fusion in 2 cases only; therefore, we can confirm that NTRK gene fusions emerge in a only a small fraction (approximately 2%) in contradistinction to other rare paediatric neoplasms, including secretory carcinoma of the breast (15), congenital mesoblastic nephroma (16) and infantile fibrosarcoma (17) where NTRK fusion is characteristic and often disease defining. Suurmeijer et al. (18) reported a series of seven NTRK3-rearranged soft tissue sarcomas as a morphologically diverse group of tumors with variable histological and immunohistochemical findings. Similarly, the morphological features of the two positive cases of our study, together with the demonstration of the NTRK rearrangement, lead to re-classify these previously not otherwise specified undifferentiated soft tissue sarcoma into the emerging category of NTRK-rearranged neoplasms. Therefore, the histological phenotype seems to play an essential role in the identification of sarcomas that are worthy candidates for immunohistochemical and/or molecular testing. As in other tumor types, URCS of soft tissue and bone and TUD represent a challenging field in NTRK fusion detection due to the rarity of fusion events (19). Several factors can explain the low positive predictive value of the IHC screening in our series. First, the pan-Trk antibody is not optimized to distinguish between wild-type protein expression and expression of chimeric fusion Trk proteins. Hechtman et al. (20) defined the pan-Trk immunohistochemistry as an efficient and reliable screen method to detect NTRK fusions. Our present series suggests that TRK can be used to direct further molecular investigation, but detection of expression is not specific to a fusion event. Thus, specificity is 20% (2 of 10 fully evaluable cases). Various factors can influence the reliability of the IHC results and correlation with molecular characterization. Certainly, histological type of tissue analyzed is increasingly evident. This study illustrates that specificity is lower in sarcomas compared to other tumour types, perhaps due differentiation in tumours of mesenchymal origin, such as neuronal (or less likely in this case, myogenic). In these and tissue types, pan-Trk antibody is known to be limited in terms of detection accuracy (21). This suboptimal specificity of IHC for fusion identification, with the antibody marking expression of wild-type Trk protein as emerged in this study, had already been noted in previous work experiences for other cancers with variable myogenic differentiation, including uterine leiomyosarcoma (22), rhabdomyosarcoma (23), and desmoplastic small round cell tumour (3). Therefore, sarcomas likely due to its tissue differentiation characteristics, can reasonably be considered a tumour type where Trk IHC lack specificity. Thus, the use and the interpretation of the IHC must be carried out with some caution, reserving primary role to the molecular testing, preferably RNA-based methods to detect NTRK gene fusions in sarcomas. This appears to be particularly important when cytoplasmic staining is present in the absence of nuclear, peri-nuclear, or membranous staining. Both NTRK fusion positive cases in our study showed a pan-Trk staining with a nuclear pattern, strengthening the IHC reliability for NTRK fusion in sarcomas, when a nuclear distribution pattern is observed. Distinct staining patterns seem to relate to the subcellular localization of fusion partner; equivalent staining patterns have been reported as well as for several of the same NTRK partner genes when they are fused to other kinases (20). The main reason of the specific staining pattern emerged in our case series may be reasonably tracked in the subcellular localization of the fusion partner; ETV6 gene encodes a transcription factor, which localizes to the nucleus. However, despite the high rate of specificity and sensitivity (24) of the molecular methods used to detect NTRK fusion in both clinical practice and research, RNA-based techniques are not free of obstacles. The main limiting factor emerged in our study was the quality or quantity of RNA available for testing, specifically in older specimens, due to degradation in formalin-fixed, paraffin-embedded tissue whilst having a larger molecular dataset would offer further insight about the sensitivity of the IHC screening. This limit predominately affects retrospective studies, when old samples are included by necessity but may be less of a barrier in daily diagnostic practice. In our study we did not detect Ewing sarcoma with distinct NTRK and EWSR1 fusions. By the clinical point of view, ETV6-NTRK3 fused sarcomas were reported as aggressive tumours, regardless of the grade of cytological atypia (18,25); we described two neoplasms with ETV6-NTRK3 fusion showing an indolent course, chemosensitive and without any signs of recurrence or metastatic spread, in contrast with what previously reported, so that the fusion partner does not seem a potential marker to predict prognosis. Further studies are needed to explore the significance of NTRK in this setting.

## Data Availability

The datasets presented in this study can be found in online repositories. The names of the repository/repositories and accession number(s) can be found in the article/supplementary material.

## Abbreviations

NTRK, neurotrophic tyrosine kinase receptor; URCS, undifferentiated round cell sarcomas; USTS, undifferentiated soft tissue sarcomas; IHC, immunohistochemistry; FFPE, formalin-fixed paraffin-embedded; NGS, next-generation sequencing; PCR, polymerase chain reaction; FISH, fluorescence *in-situ* hybridization; ESMO, European Society for Medical Oncology; WHO, World Health Organization; AJCC, American Joint Committee on Cancer.
